# Release of sterile *Aedes aegypti* mosquitoes: chilling effect on mass-reared males survival and escape ability and on irradiated males sexual competitiveness

**DOI:** 10.1038/s41598-023-30722-9

**Published:** 2023-03-07

**Authors:** Génesis Alejandra Sánchez-Aldana-Sánchez, Pablo Liedo, J. Guillermo Bond, Ariane Dor

**Affiliations:** 1 El Colegio de la Frontera Sur (ECOSUR), 30700 Tapachula, Chiapas Mexico; 2 Centro Regional de Investigación en Salud Pública, Instituto Nacional de Salud Pública (CRISP, INSP), 30700 Tapachula, Chiapas Mexico; 3 Consejo Nacional de Ciencia y Tecnología (CONACYT), commissioned to El Colegio de la Frontera Sur (ECOSUR), 30700 Tapachula, Chiapas Mexico

**Keywords:** Animal behaviour, Entomology, Animal biotechnology

## Abstract

In the sterile insect technique, it is important to measure the impact of mass-rearing and handling of sterile males to allow a successful control of the target wild population. This study evaluates the effect of pre-release chilling on the survival, escape ability, and sexual competitiveness of male *Aedes aegypti*. To determine survival and escape ability, mosquitoes were chilled at 4 °C using four different treatments of either one exposure (25 min) or two consecutive exposures (25 + 25 min, 25 + 50 min, 25 + 100 min). For sexual competitiveness, two different treatments were evaluated, chilling for 25 min once and twice. Results showed that the longest exposure to chilling caused a significant reduction of survival time, from 67 to 54 days. Escape ability was reduced by the first chilling from 25 to 7% and with the second chilling, it was reduced from 30 to 24% in the control to 4.9, 2.0 and 0.5% for 25, 50 and 100 min, respectively. Sexual competitiveness index was reduced from 1.16 in the control, to 0.32 and − 0.11 for treatments involving one and two chilling periods, respectively. It is recommended to increase the chilling temperature and reduce the exposure time to reduce the harmful effects on sterile males.

## Introduction

The mosquito *Aedes aegypti* predominates in urban and suburban habitats and is one of the main agents responsible for massive dengue outbreaks^1–3^. It is also the main vector of Chikungunya^4^, Zika^5^, and yellow fever virus^6^, all of which are arboviruses that have caused major epidemics across the globe. *Aedes aegypti* is a major source of concern for tropical and subtropical regions across the world due to its vector competence and efficient dispersal^7^. Disease prevention strategies have focused on vector control because there are as yet no commercial treatments or vaccines available against dengue, chikungunya, or Zika. Current conventional preventive methods include solid waste management (recycling and garbage disposal) and the elimination of natural and artificial breeding sites. Larvae are controlled by the application of larvicides in permanent water containers inside and outside of residences, whereas adults are targeted by residual insecticide spraying in buildings and residences and by emergency space spraying in areas where the diseases have been reported^8,9^.

However, the increasing incidence of these viruses indicates that these measures are insufficient. The criticality of the situation has propelled the search and development of new methods and control strategies with a lower environmental impact^10^, for instance the use of genetically modified mosquitoes^11,12^, *Wolbachia*-based mosquito population control^13^, and the sterile insect technique (SIT)^14–16^. In our research, we mainly focus on SIT, a method that relies on the mass-rearing, sterilisation, and release of sterile male to reduce the birth rate of the target population^17^. The mating of sterile males with fertile females results in the deposition of inviable eggs, and consequently, leads to a decline in the target population. SIT has been successfully applied in the control of agricultural pests and disease-transmitting vectors^17,18^. Inspired by the promising results of SIT, the World Health Organization (WHO) and the Food and Agriculture Organization of the United Nations/International Atomic Energy Agency (FAO/IAEA) endorse the use of SIT as a tool to suppress the dispersion of disease vector mosquitoes^9^. The technique is already being validated in pilot projects in various countries^19,20^.

In Mexico, a pilot project was launched in 2016 to validate the efficiency of SIT as a means to suppress the *Ae. aegypti* populations^21^. Mass-rearing of male mosquitoes was initiated in 2018 at the mass-rearing facility of the Centro Regional de Investigación en Salud Pública – Instituto Nacional de Salud Pública (CRISP-INSP) located in Río Florido, municipality of Tapachula, Chiapas. Releases of *Ae. aegypti* sterile male mosquitoes were carried out at the rural village Hidalgo (14°53′4″ N, 92°21′28″ W), comparing ground and aerial releases. A higher recapture rate was recorded for ground releases^22^.

Different chilling protocols were followed for the evaluation of ground and aerial releases by Marina et al.^22^. In both cases, irradiated male pupae were placed in emergence chambers. In the case of ground releases, emergence chambers with 2-d old adult males were chilled at 4 ± 1 °C for 15 min to immobilise the males as the pupal container was separated from the upper chamber that contained the adults. After three days, the adults were transported in containers to the release site and were released. For the aerial releases, the adults were additionally exposed to a second chilling period of 15 to 25 min at 4 ± 1 °C. Following the second chilling, the mosquitoes were transported for a duration of 10 min in a portable cooler (6 ± 1 °C) to the release sites where they were loaded into a drone for release^22^.

The rationale behind SIT is that large numbers of sterile male mosquitoes are released to outcompete the wild fertile males and mate with wild fertile females. Therefore, the released mosquitoes have to adhere to the highest quality standards. Sterile male mosquitoes must be able to tolerate specific handling, transport, and release conditions^23^ and, once in the field, disperse in search of females and mate^24^. Several authors have demonstrated that chilling affects the survival rate of mosquitoes. Survival rate of male *Anopheles arabiensis* maintained at 2 °C for 24 h was lower than for those chilled at 4 °C^25^. A similar observation was reported for a Brazilian *Ae. aegypti* strain: chilling males at 0 °C for 2 h decreased their survival rate, while a temperature of 8 °C adversely impacted their ability to fly out of a flight test device (escape ability) in comparison with a control at 25 °C^26^. Other authors confirmed that escape ability was negatively affected by chilling at 4 °C for 2 h, as well as by irradiation^27^.

How much does pre-release chilling affect the quality of sterile male *Ae. aegypti* mosquitoes? And how does this impact ground and aerial releases? In the present study, the effect of chilling within the context of ground and aerial releases was evaluated on the survival and escape ability of mass-reared *Ae. aegypti* males, and on the sexual competitiveness of sterile males. For survival and escape ability, we compared four treatments: 1) a 25-min chilling (1_25), 2) two chillings, the first for 25 min and the second for 25 min (2_25), 3) two chillings, one of 25 min and the next for 50 min (2_50), and 4) two chillings, one for 25 min and the second for 100 min (2_100). For sexual competitiveness we compared two treatments, 25 min chilling once (Ho_1_25), and 25 min chilling twice (Ho_2_25).

## Results

### Effect of chilling on non-irradiated male survival

**Figure 1:**
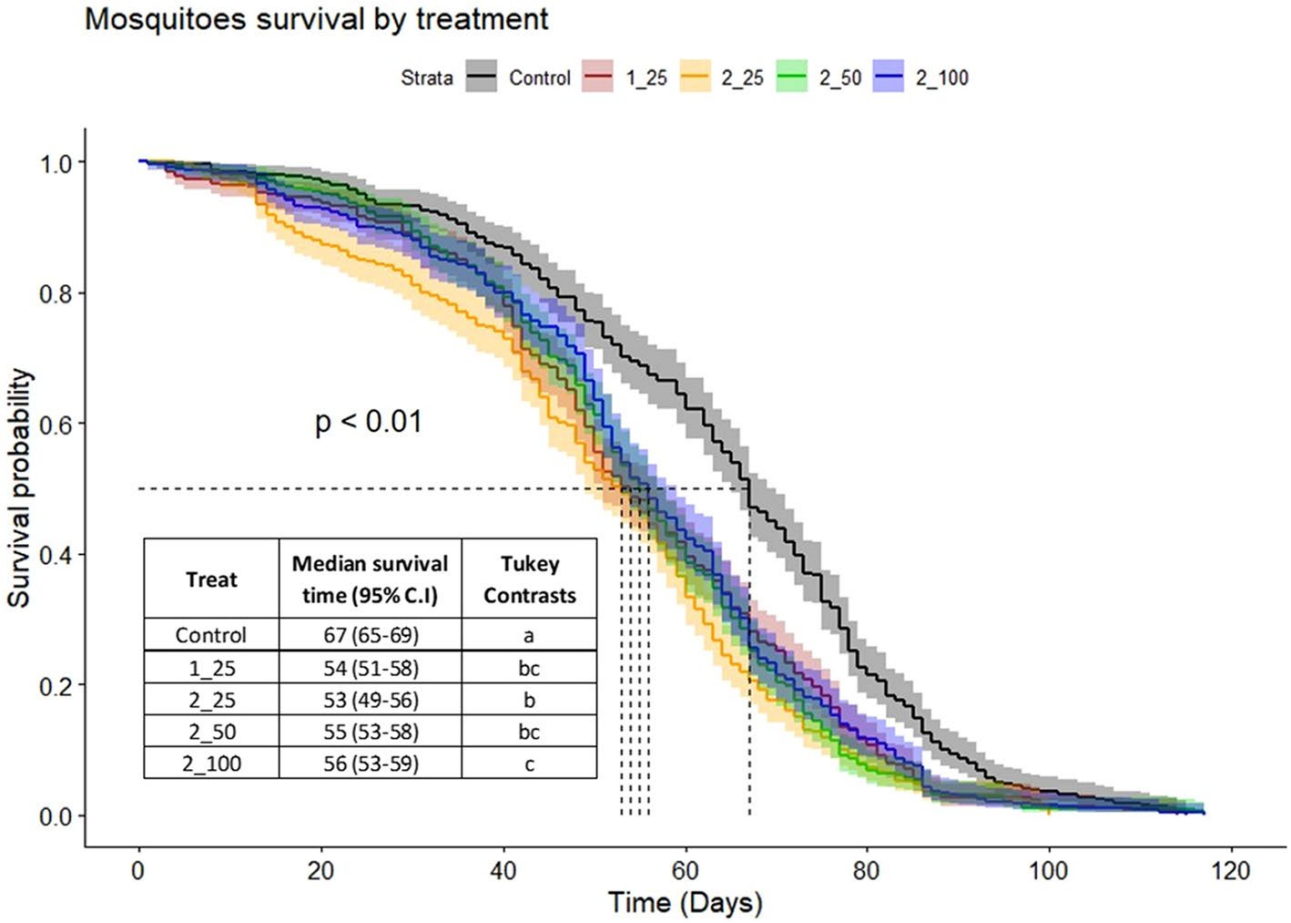
Kaplan–Meier survival curves of flyer GDS *Aedes aegypti* males after different chilling processes. Kaplan–Meier survival curves for *Aedes aegypti* males after different chilling processes: control, no chilling; 1_25, one exposure to chilling, 25 min; 2_25, two exposures to chilling, 25 + 25 min; 2_50, two exposures to chilling, 25 + 50 min; and 2_100, two exposures to chilling, 25 + 100 min. Jagged lines represent the mean survival times for each treatment. Treatments sharing a common letter indicate non-significant differences, according to Tukey test.

Survival curves were fitted to daily adult male mosquito mortality (*N* = 1996). The mean survival time of male *Ae. aegypti* was significantly reduced in the chilling treatments (54 to 56 days) compared to the control (67 days) (Fig. 1).

### Effect of chilling on non-irradiated male escape ability

**Figure 2:**
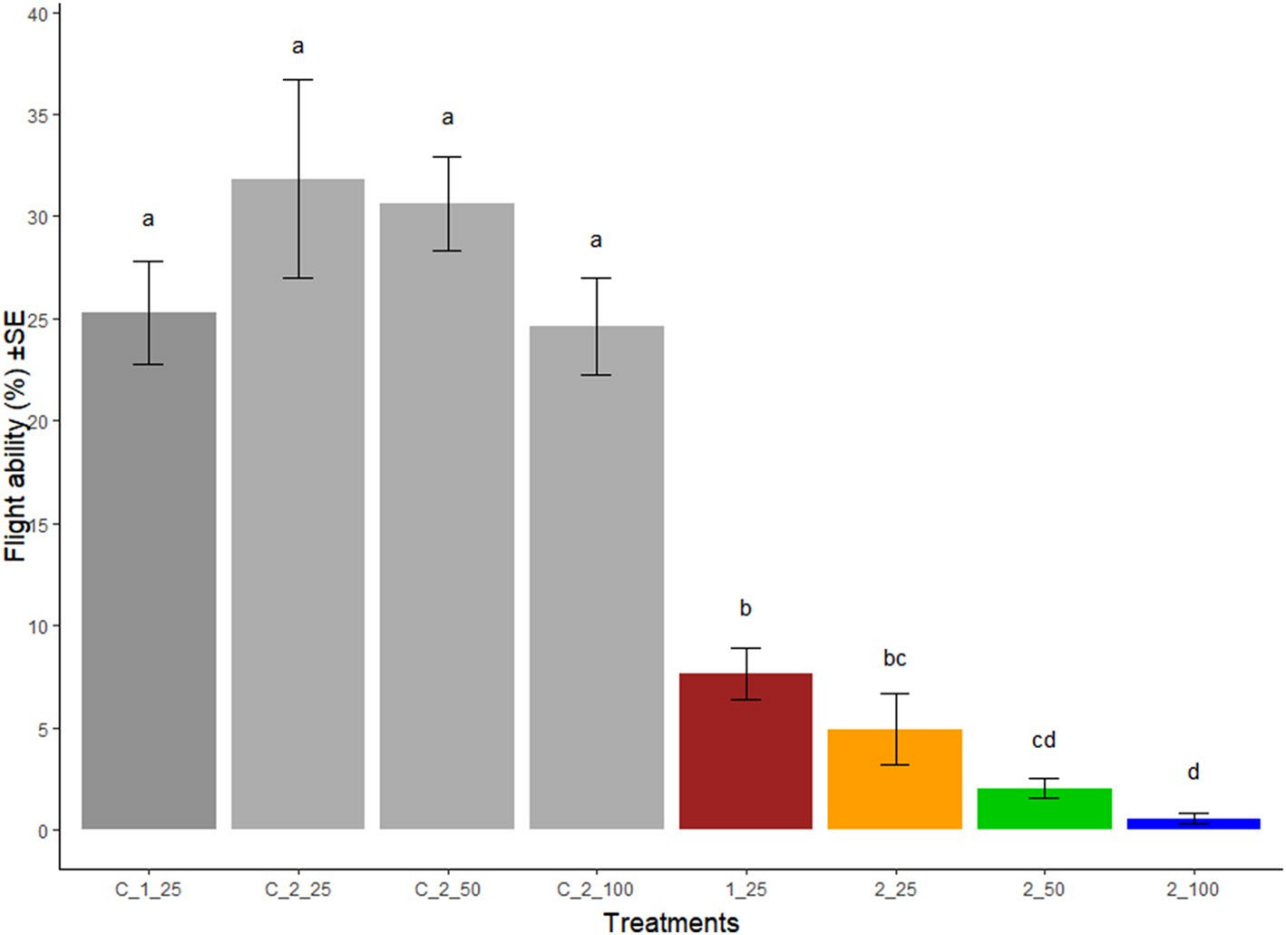
Percentage of flyer GDS *Aedes aegypti* males (males that escaped from the flight test device) after different chilling processes. Percentage of flyer male mosquitoes (males that escaped from the flight test device) ± standard error (SE) after different chilling processes (11 replicates per treatment). Coloured bars correspond to 1_25 (one exposure, 25 min), 2_25 (two exposures, 25 + 25 min), 2_50 (two exposures, 25 + 50 min), and 2_100 (two exposures, 25 + 100 min). Grey bars represent the corresponding controls for each treatment. Treatments sharing a common letter indicate non-significant differences, according to Kruskal–Wallis test.

**Figure 3:**
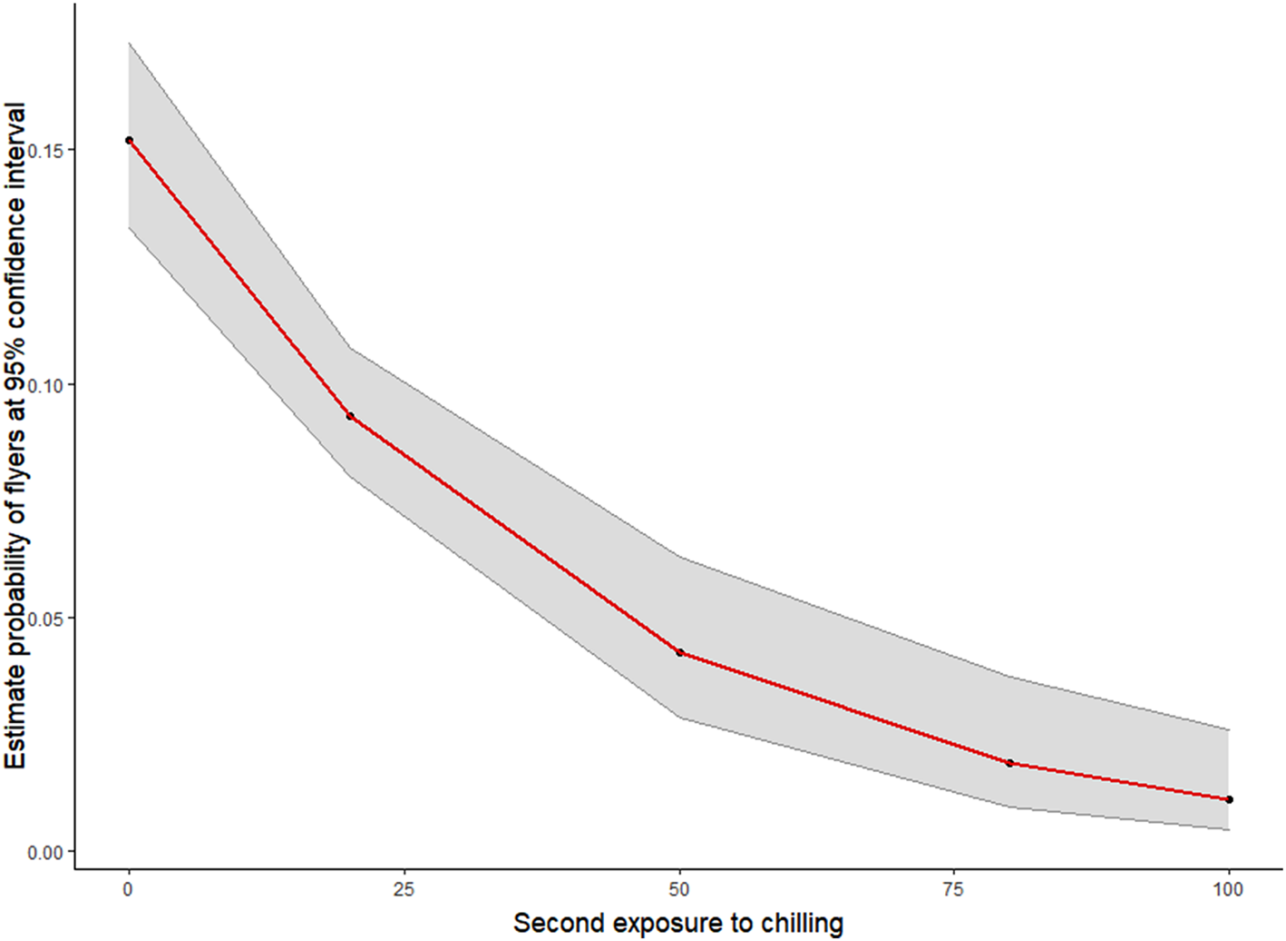
Model of the effect of second chilling on escape ability (ability to fly out of a flight test device) of GDS *Aedes aegypti* males. Linear regression model for the escape probability after a second exposure to chilling temperatures (25, 50, and 100 min).

Escape ability of male *Ae. aegypti* was significantly reduced as the exposure time to chilling was prolonged (Fig. 2). When only one chilling treatment was applied (1_25), 7.6% of the males were able to escape from the flight test device, compared to 25.3% in the corresponding control. When two chilling treatments of 25 min each (2_25) were applied 4.9% individuals were able to escape, compared to 31.8% in its control. In treatment 2_50, 2% escaped compared to 30.7% in the control. In treatment 2_100 only 0.5% escaped compared to 24.6% in the control. The generalised linear model indicated that extension of the second chilling period decreased the probability that males would succeed in flying through the flight test device (Fig. 3).

### Effect of chilling on the sexual competitiveness of irradiated males

#### Egg hatching rate

**Figure 4:**
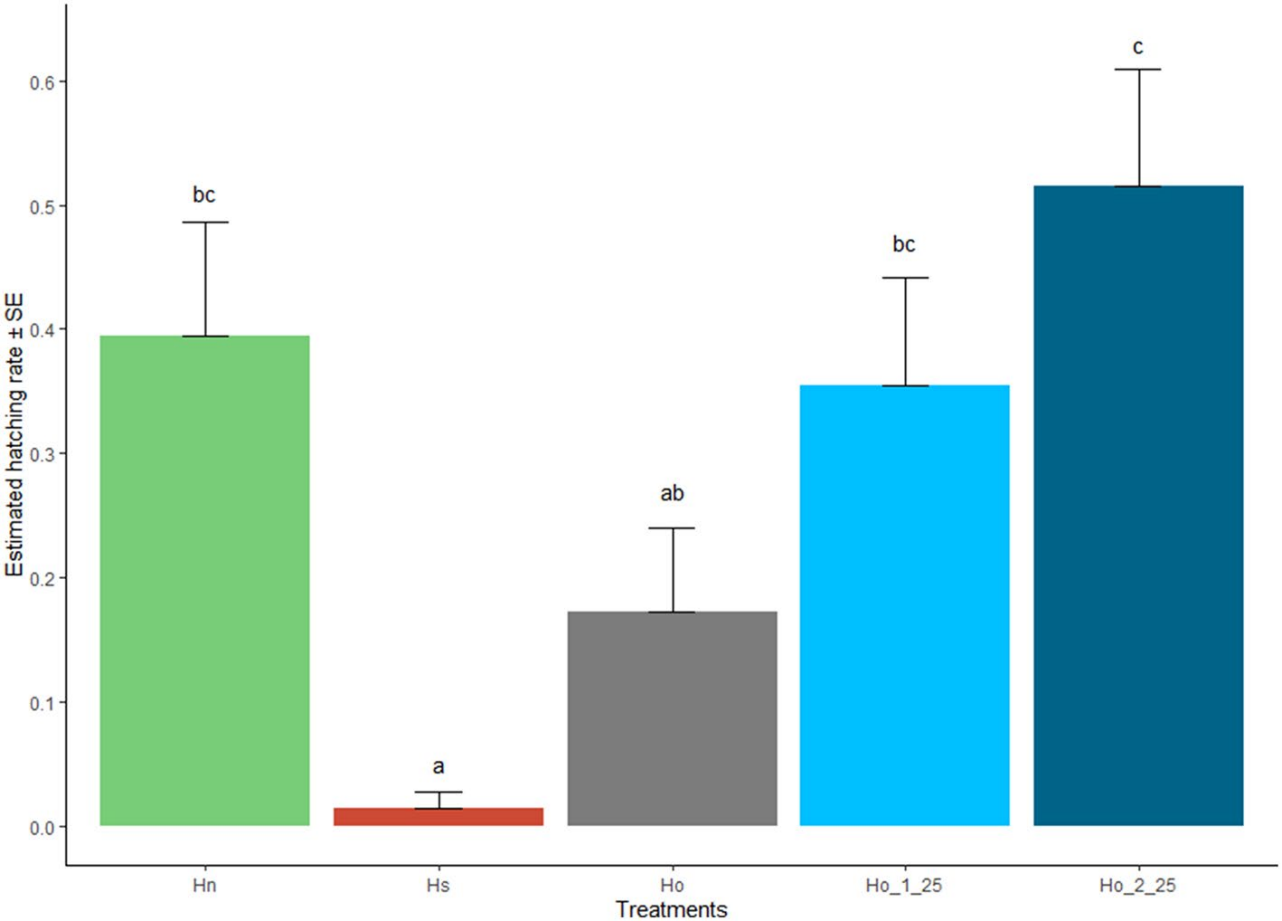
Egg hatching rate of GDS *Aedes aegypti* after different chilling processes. Egg hatching rate ± standard error (SE) of two controls and three treatments. Controls: *Hn*, 50 fertile females + 50 fertile males; and *Hs*, 50 fertile females + 50 sterile males. Treatments: *Ho*, 50 fertile females + 50 fertile males + 50 sterile males, no chilling; *Ho_1_25*, 50 fertile females + 50 fertile males + 50 sterile males, chilled at 4 °C for 25 min; and *Ho_2_25*, 50 fertile females + 50 fertile males + 50 sterile males, chilled at 4 °C for 25 min (twice). Treatments sharing a common letter indicate non-significant differences, according to Kruskal–Wallis test.

The egg hatching rate was 37.66% for fertile females that mated with fertile males (fertile control *Hn*). Egg hatching was significantly lower when fertile females were mated with sterile males (sterile control *Hs*) than when they mated with fertile males (*Hn*). Also, a significantly lower egg hatch was observed for non-chilled (*Ho*) compared to twice-chilled irradiated males (*Ho_2_25*, Fig. 4).

#### Effect of chilling on the sexual competitiveness and induced sterility of males

**Figure 5:**
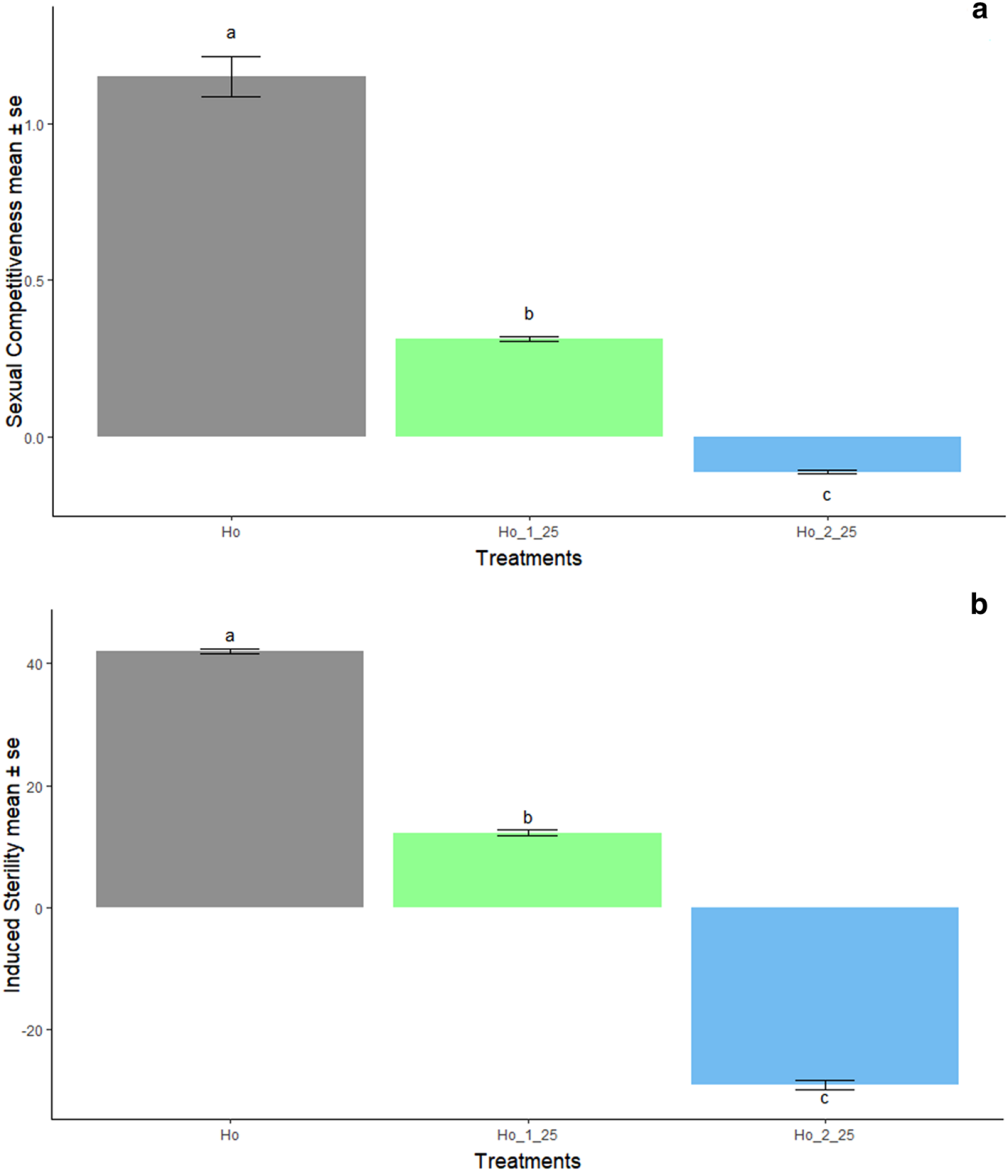
Chilling effect on sexual competitiveness and induced sterility in the GDS *Aedes aegypti*. (**a**) Chilling effect on sexual competitiveness: mean values of the GDS *Aedes aegypti* male sexual competitiveness ± standard error (SE). (**b**) Chilling effect on induced egg sterility percentage: Mean values of the GDS *Aedes aegypti* female sexual induced egg sterility ± standard error (SE). Treatments sharing a common letter indicate non-significant differences, according to Tukey test.

The sexual competitiveness index of non-chilled irradiated males (*Ho*) was 1.16. The competitiveness value was decreased by exposure to chilling temperatures: 0.32 and the negative value -0.11 for treatments involving one (*Ho_1_25*) and two (*Ho_2_25*) periods of exposure, respectively. The percentage of induced sterility was 41.35% in treatment *Ho* and decreased to 12.40% and -29.30% in the chilling treatments, *Ho_1_25* and *Ho_2_25*, respectively (Fig. 5).

## Discussion

Our results show that chilling has a significant effect on the performance of sterile mosquitoes, and the more frequent and longer chilling times, the stronger the adverse effects.

Regarding survival, our data from the control treatments were in agreement with Bond et al.^28^, which suggests that the quality characteristics of the Genetically Diverse Strain (GDS) has remained stable over this 5-year period. Male survival was negatively affected, particularly when the exposure time of the second chilling period was increased to 100 min. This observation agrees with Gómez et al.^29^, who evaluated the effect of different temperatures (4, 7, 10, and 14 °C) and exposure times (60, 90, and 120 min), and found that exposure to a temperature of 4 °C for 120 min affected male *Ae*. *aegypti* survival rate. Similar observations were made by Culbert et al.^23^, who evaluated different chilling temperatures (4, 6, 8, or 10 °C) for 1, 4, 8, and 24 h, and demonstrated that extending the exposure time at 6 °C had a negative impact on male *Ae*. *aegypti* survival rate. Our results, however, are at variance with Culbert et al.^26^, who evaluated different exposure temperatures (0, 4, 8, and 10 °C) for 2 h, and found that only the survival rate of males exposed to 0 °C was reduced. The disagreement might be explained by a difference in exposure time (2 h in Culbert et al.^26^), compared to 25 min and 100 min in our study).

Also, escape ability in our controls was very low (between 24.6 and 31.8%) in comparison with Dor et al.^30^, who reported a 50% escape rate using the same device. We suspect that the 10-month interruption in the rearing of the GDS colony (due to the COVID-19 pandemic) might have affected this biological feature. However, escape from the selected flight test device (80 cm tall, 2 cm diameter) proved challenging and allowed us to demonstrate the negative effect of chilling temperatures on male mosquitoes. Escape ability was affected by the first exposure to chilling temperatures and was further reduced as exposure time of the second exposure increased. These findings are in agreement with Gómez et al.^29^, who showed that the escape rate of male mosquitoes from the FAO/IAEA recommended device was affected by exposing the males to a temperature of 4 °C for 60 min. Our results are also in agreement with the observations by Culbert et al.^26^ and Maïga et al.^27^ who, using the same device, demonstrated that exposure to a temperature equal or less than 4 °C for 2 h adversely affected escape ability. None of these authors, however, evaluated the impact of varying the time of exposure to low temperatures. Therefore, to confirm the results, it is necessary to directly compare escape ability trials in the flight test device described by Dor et al.^30^ and in the one recommended by the FAO/IAEA.

Egg hatching data indicate that chilling treatments lead to higher hatching rates, suggesting that sterile males become less sexually competitive. Therefore, fertile females will be more likely to copulate with fertile males, leading to increased egg hatch. The presence of spermatozoa in the spermathecae of females that had been in contact with once as well as twice chilled sterile males should be verified by direct observation, calculating the percentage of insemination^31^.

Sexual competitiveness and induced sterility values for non-chilled sterile males were higher in our results than in Bond et al.^31^ for the same strain. This indicates that irradiated males were competitive with fertile males at the time of the experiment, and that the first exposure to chilling temperatures negatively affected the sexual competitivity of irradiated males. A second exposure to chilling temperatures impacted this parameter even more and gave negative values. These findings strikingly show that fertile males copulate more when they compete with twice-chilled sterile males compared to the fertile control (*Hn*). This is likely due to the natural variation in the response of fertile males to fertile females in the absence of sexual competition from other males. Further investigations should clarify this point.

In successful SIT pilot projects with *Aedes albopictus* it was found that the sexual competitiveness index was greater than 0.2^32^. This suggests that the chilling of males for ground release can be an acceptable alternative, whereas the chilling of males for aerial release adversely impacts the efficiency of SIT.

For the implementation of SIT to be successful, mass-rearing, managing, sterilisation, and transport should affect the quality of the produced sterile mosquitoes as little as possible. Therefore, and based on our findings, we suggest that sterile males are immobilized at temperatures higher than 4 °C, and to investigate which time–temperature combination will be the least harmful to them. Temperatures between 5 and 10 °C for less than 3 h were recommend^33^, and it was suggested immobilising the males at 7-10 °C for a short period^26^, moreover, Ernawan et al.^34^ advise to use a temperature of 7 °C for a period that does not exceed 24 h. Within the context of our study, it would be recommended to evaluate the exposure of the mosquitoes to temperatures in the range 4-10 °C for 10–25 min, and to determine the effect on survival, escape ability, and sexual competitiveness under semi-field conditions using standardised protocols, which would allow comparison with other studies. Also, it would be recommended to make improvements in the design of the emergence chamber, so that the first chilling process can be eliminated. Similarly, it would also be necessary to reassess the protocols used to load the drone with sterile males. A climate chamber, for instance, would allow a quick and effective immobilisation of the mosquitoes^35^. Alternatively, it could be advisable to assess if after a certain time after chilling, sterile mosquitos might recover their sexual competitiveness and escape ability, since Gómez et al.^29^ demonstrated that *Ae. aegypti* males exhibited better escape ability after a recovery period of 24 h.

To conclude, our study shows that the chilling procedure described by Marina et al.^22^ affected the survival and escape ability of mass reared *Ae. aegypti* males and the sexual competitiveness of irradiated males. Since irradiation does not adversely affect the performance of sterile mosquitoes^32,36,37^, it is expected that the effect of the chilling process will be similar, or even higher, on irradiated males than on non-irradiated mass-reared males. Our results clearly show that the stress caused by two exposures to temperatures of 4 °C for 25 min each, adversely affects males’ performance. Also, because of its impact on escape ability and sexual competitiveness, it is not advisable to prolong the exposure time of the second chilling period to more than 25 min (at 4 °C). This could in part explain why less released sterile males were recaptured after aerial releases than after ground releases^22^.

## Methods

### Mosquito strain and rearing: colony maintenance and laboratory trials

The genetically diverse strain (GDS) of *Ae. aegypti* mosquitoes used in the experiments was originally collected from twelve localities along the coast of Chiapas, Mexico^28^. The strain (64th generation) was maintained for seven generations under controlled conditions of temperature (27 ± 2 °C) and relative humidity (RH 80 ± 5%), under a 12-h light/dark cycle. Larvae were reared at a density of 1.25 larvae/mL in plastic trays (45.7 × 31.8 × 5.4 cm) containing 2000 mL filtered water (100 µm filter) and were fed a 4% liquid suspension of a certified laboratory rodent diet (LabDiet 5001; PMI Nutrition International LCC, St. Louis, MO), as previously described by Bond et al.^28^. Due to the COVID-19 pandemic, mosquito field releases planned for April 2020 were suspended and colony maintenance at the mass-rearing facility was discontinued. The GDS was reactivated in January 2021, after having been dormant for 10 months. Three months were needed to obtain sufficient numbers of eggs for the laboratory experiments.

Pupae were separated by sex using a glass plate separator (John W. Hock, model 5412, Gainesville, Florida, USA). The mechanical separation is based on body size, as male pupae are smaller than female pupae for a given quantity of diet. Next, pupae (500 males and 1500 females) were placed in containers within a BugDorm cage (30 × 30 × 30 cm cage, acrylic frame with nylon mesh panels; BugDorm 1, Taichung, Taiwan) and left to emerge. Adult populations were maintained and reared under controlled conditions of temperature (25 ± 2 °C) and RH (60 ± 10%) and were provided with a 10% sugar solution^38^. To obtain eggs, females were provided with a sheep blood meal five days after emergence and seven days after the first feeding (two gonotrophic cycles). Eggs produced by the brood-stock were hatched for use in the experiments according to the previously described protocol. Individuals at the pupal stage were transferred to emergence chambers, as described by Marina et al.^22^.

### Chilling of male mosquitoes

Male mosquitoes were chilled following two different protocols described by Marina et al.^22^, but chilling time was increased from 15 to 25 min to harmonise the implementation of the new methodologies with the standard operating procedures of the mass-rearing facility (standard chilling time: 15 to 25 min). Mosquitoes were chilled in a conventional upright refrigerator (General Electric, TA10ZL). Preliminary experiments had shown that the mosquitoes were immobilised after 20 min at 4 °C.

#### Chilling protocol for ground releases

Emergence chambers with 48 h post-emergence males were chilled at 4 ± 1 °C for 25 min in a refrigerator to immobilise them in the upper chamber compartment as the pupal container was removed. Males remained in the upper chamber and were provided with a cotton pad moistened with 10% sucrose at 26 ± 2 °C until use in laboratory experiments.

#### Chilling protocol for aerial releases

The upper chamber was chilled for a second time 48 h after the first exposure. It was chilled at 4 °C for different periods of time (25, 50, or 100 min), depending on the type of treatment.

### Effect of chilling on non-irradiated male survival

Four treatments and one control were set up. The first treatment was chilled only once at 4 °C for 25 min (1_25), whereas the three other treatments were first chilled at 4 °C for 25 min and then again at 4 ± 1 °C for different exposure times: 25 min (2_25), 50 min (2_50), and 100 min (2_100). The control was not exposed to chilling temperatures. Following these treatments, males remained in the upper chamber to recover for one hour. Mosquitoes were then moved to BugDorm cages and daily mortality was recorded until all adults were dead. Each treatment (including the control) consisted of 100 males and was replicated four times. Each replicate corresponded to a different rearing batch.

### Effect of chilling on non-irradiated male escape ability

Escape ability was measured using a flight test device (80 cm tall, 2 cm diameter) that was previously described by Dor et al.^30^ as the most challenging for males to escape from. Each treatment (1_25, 2_25, 2_50, and 2_100) was evaluated with its corresponding control (C_1_25, C_2_25, C_2_50, and C_2_100). All treatments were exposed to chilling for the first time at 2 days after male emergence. For logistical reasons, the second exposure was applied at different days: 3 days post-emergence for 2_25 and 2_50, and 4 days post-emergence for 2_100. After the last chilling, males remained in the emergence chamber to recover for one hour before being transferred to the flight test device’s loading chamber with the help of a mouth aspirator. For each treatment, 50 males were placed in the loading chamber. Eleven replicates were performed, each from a different rearing batch.

The number of males that managed to escape from the device after 2 h was recorded. The escape rate was calculated by dividing the number of escaped mosquitoes by the total number of mosquitoes placed in the loading chamber.

### Effect of chilling on the sexual competitiveness of irradiated males

Batches of 2500 pupae were separated 24 h before adult emergence. From each batch, 500 male pupae were selected and transferred to plastic trays (10.5 cm diameter) containing 50 mL filtered water for irradiation. Irradiation was carried out with a dry storage irradiator (Gamma Beam GB-127, serial number IR-226; Nordion, Ottawa, Canada) with ^60^Co source (activity 14416 Ci) at the MOSCAFRUT (SENASICA-IICA) facility, Metapa de Domínguez, Chiapas, Mexico (14° 49′ 49″ N, 92° 11′ 44″ W). Pupae received a dose of 50 Gy, which induces a sterility of 99.4%^38^. Irradiated pupae were then placed in emergence chambers at 26 ± 2 °C for 48 h.

Sexual competitiveness was evaluated under controlled conditions of temperature (26 ± 2 °C) and RH (80 ± 5%), under a 12-h light/dark cycle. Mosquitoes were maintained in cages (30 × 30 × 30 cm) and randomly distributed over five different treatments: *Hn* (fertile males control), with 50 fertile females and 50 fertile males; *Hs* (sterile males control), with 50 fertile females and 50 sterile males; *Ho*, with 50 fertile females, 50 fertile males, and 50 sterile males (non-chilled); *Ho_1_25*, with 50 fertile females, 50 fertile males, and 50 sterile males chilled at 4 °C for 25 min (48 h post-emergence) and allowed to recover for 24 h; and *Ho_2_25*, with 50 fertile females, 50 fertile males, and 50 sterile males exposed twice to a chilling temperature of 4 °C for 25 min (at 48 h and 72 h post-emergence). After the second exposure to chilling, irradiated males were allowed to recover for 1 h. In all treatments, males were gently released into the cages, followed 1 h later by the release of females, as previously described^31^. After 24 h, females were removed and transferred to another cage (30 × 30 × 30 cm) and fed with ovine blood for 1 h using a parafilm-membrane feeding system (PM992, Bemis Company, Inc.). Eggs were collected in oviposition traps (500 mL transparent plastic container, 11 cm diameter × 9 cm height, containing 200 mL of water and a 32 × 5 cm filter paper strip for oviposition) that were placed in each cage at 72 h after the first feeding. After 48 h, the containers were removed from the cages, the number of eggs on the paper strips was recorded, and eggs were placed on humid bedding. Females were given a second feeding at five days after the first blood meal, and eggs were collected as before. The number of hatched and unhatched eggs was recorded, and eggs were hatched in a 150 mL glass container with water (38 °C). After 48 h, the number of hatched and unhatched eggs as well as the number of larvae was counted, which allowed us to determine egg production and hatching rate, and to estimate induced sterility.

The male mating competitiveness index (*C*) was calculated using Fried’s equation^39^:

$$C = (Hn - Ho)/(Ho - Hs)*(N/S)$$

where *Hn* represents the hatching rate when only fertile individuals were involved (1 fertile male:1 fertile female); *Ho* is the observed hatching rate for the ratio 1 fertile male:1 fertile female:1 sterile male. Three options are possible for de sterile males: non-chilled (*Ho*), chilled once (*Ho_1_25*), and chilled twice (*Ho_2_25*); *Hs* is the hatching rate of eggs from females that mated with sterile males (1 sterile male:1 fertile female); *N* corresponds to the number of fertile males at the beginning of the experiment (50); and *S* is the number of sterile males (50). Induced sterility (*IS*) was then calculated using the equation:

$$IS = (1 - (Ho/Hn)) * 100$$

### Statistical analysis

Survival curves were generated using the Kaplan–Meier method and fitted with the Cox proportional-hazards model. Pairwise comparisons were performed using Tukey’s test. Escape ability data were analysed using the Kruskal–Wallis test and Fisher's least significant difference test to compare and group the different treatments. Probability of success was determined using a generalised linear model with binomial response for the second chilling three different times (25, 50 and 100 min). The negative impact of influential points was mitigated by using binary Dummy variables^40^. The hatching rate was estimated with a beta-binomial generalised linear mixed model (GLMM).The sexual competitiveness index was calculated using Fried’s equation^39^, data were reiterated 5000 times to obtain variability, finally mean competitiveness and induced sterility values were compared by the Tukey test. Survival data were analysed using the survival package (R package version 3.4–0; Therneau T 2022) and the drawing of survival curves was performed using the survminer package (R package version 0.4.9; Alboukadel Kassambara, Marcin Kosinski and Przemyslaw Biecek 2021). Escape ability and sexual competitiveness data were analysed using the statistical software package R (version 4.1.2; R Core Team 2021, Therneau 2022). Comparison of the GLMM treatments was analysed with the multcomp package. (R package version 1.4–20; Torsten Hothorn, Frank Bretz, Peter Westfall, Richard M. Heiberger, Andre Schuetzenmeister, Susan Scheibe 2022). R script is available in Supplementary Data 2.

## Supplementary Information

Dataset S1.

Dataset S2.

## Data Availability

The datasets generated and analyzed and the R script during the current study are available in the Supplementary Data 1 and 2.
